# The Collapse of a “College”

**Published:** 1900-05

**Authors:** 


### The Collapse of a "College."

**SIC TRANSIT.**

READERS of the JOURNAL will recall our account of the chartering by the State of Texas of the New York Medical Callege, at San Antonio, and the statement we made that under our laws diplomas issued by the concern were lawful, and equivalent to a license to practice. This being the case, we asked: "How are these fellows to be gotten at?" and said: "Wait and see." The denouement has come. Representatives of the medical press of the city and of the West Texas Medical Society conferred with the Attorney-General and he said if we would get the "facts" he would do the rest. The facts were forthcoming; facts demonstrating that a merest pretext of teaching medicine was gone through with, and that the concern was virtually selling diplomas. For illustration: The West Texas Medical Society had the colored janitor of the Hicks building to matriculate. He could scarcely read and write. He went to the college every day and was "taught," he says, by one man, orally. In seventeen days he was "graduated," and given a diploma in dog-Latin, with thirteen names to it, representing thirteen branches of medicine and surgery. The "Professor of Obstetrics and Diseases of Women," we are informed, whose name was signed to the diploma, was the seven-year-old daughter of the proprietor, the boss, who was running the mill. The editor of this JOURNAL caused several letters of inquiry to be addressed to the "College," and the answers sent to the JOURNAL. They were highly incriminating, and were all promptly turned over to Attorney-General Smith. One letter asked the cost of getting a diploma for a friend, and the reply was "fifty dollars." The "dean," who, it seems, wrote the letter, added: "If you will throw this business into our hands we will remember you with a ten." Another doctor wrote that he had a license from an examining board, but no diploma, and that he wanted to get a diploma, and asked what it would cost him. It was offered him "under the circumstances," at $15. In one of these letters it is stated that the names to the diplomas, as professors of the different branches, are the names of "graduates" of the concern, written by

Mr. Forest, or the College, in the absence of the parties, but with their permission. The Attorney-General filed a petition in the district court at San Antonio, asking that the charter be forfeited on the grounds that it was being abused and the "college" made no defense; hence judgment went by default, and we give below the text of the decision, for which we are indebted to Attorney-General Smith. The thanks of the medical profession are due, and were, at the recent meeting of the Texas State Medical Association, tendered to Judge Smith for his intelligent and prompt action in thus removing this stigma upon the escutcheon of the State. We derive much comfort from the hope that the profession will now be able to show to the satisfaction of the Governor and the next Legislature that a "diploma from a chartered medical college" is not, or should not be, a license to practice, but is a most dangerous thing—a menace to the public health, and that our miserable medical practice act which recognizes a diploma should be at once repealed.

The following is the judgment, closing up the New York Medical College, of San Antonio, and perpetually restraining them from issuing any more diplomas. Suppose, now, that some of the victims should bring suit for swindling? How would that work?

No. 11,786.

| | |
|---|---|
| The State of Texas,<br>vs.<br>New York Medical College. | In the District Court, Thirty-seventh Judicial District, Bexar county, Texas. |

On this the 14th day of May, A. D. 1900, came on to be heard the above styled and numbered cause, and came the plaintiff, the State of Texas, by T. S. Smith, Attorney-General, and Carlos Bee, the District Attorney, and the defendant, The New York Medical College, and J. D. Forest, N. E. Forest and R. B. Forest, directors thereof, though duly cited, failed to appear, and wholly made default, wherefore it is considered by the court that the plaintiff, the State of Texas, have judgment in this cause.

It is therefore ordered, adjudged and decreed by the court, first, that the corporate charter granted by the State of Texas to the defendant, The New York Medical College, on December 5, 1899, be and the same is hereby forfeited unto the State of Texas, and that the said corporation be and the same is hereby dissolved and its said charter is declared void, and that the franchise of said corporation be extinguished, and that said defendant be ousted therefrom.

It is further ordered that the defendant corporation, its officers, agents and employes, be and they are hereby perpetually enjoined and restrained from granting or issuing diplomas, either under the guise of said corporation or otherwise, and that the said defendant corporation, its officers, agents and employes be and they are hereby perpetually enjoined and restrained from carrying on the business of the New York Medical College under and by virtue of the charter heretofore issued by the State of Texas, and it is further ordered that the said New York Medical College, its officers, agents and employes, be and they are hereby perpetually enjoined from offering for record or from having recorded any diplomas of any kind, character or description whatever, granted or issued by the said New York Medical College, or under its direction or by its authority.

It is further ordered that the plaintiff, the State of Texas, do have and recover of and from the defendant, the New York Medical College, all costs in this behalf expended, from which let execution issue.

It is further ordered, adjudged and decreed by the court that the officers of the court do have and recover of and from the State of Texas the costs incurred by it in this suit.

